# Intra-operative intra-peritoneal chemotherapy with cisplatin in patients with peritoneal carcinomatosis of ovarian cancer

**DOI:** 10.1186/1477-7819-7-14

**Published:** 2009-02-09

**Authors:** Emmanuel Guardiola, Delphine Delroeux, Bruno Heyd, Marielle Combe, Veronique Lorgis, Martin Demarchi, Ulrich Stein, Bernard Royer, Bruno Chauffert, Xavier Pivot

**Affiliations:** 1 University Hospital Jean Minjoz, Department of Medical Oncology, 25030 Besançon Cedex, France; 2 CHU Jean Minjoz, Department of surgery, University Hospital Jean Minjoz 25030 Besançon Cedex, France; 3 Department of Anaesthesia-Intensive Care, 25030 Besançon Cedex, France; 4 Department of Pharmacology, 25030 Besançon Cedex, France; 5 Anti cancer center Georges-François Leclerc, 21000 Dijon, France

## Abstract

**Background:**

Intra-peritoneal (i.p.) chemotherapy is an encouraging treatment option for ovarian cancer with peritoneum involvement in addition with intravenous (i.v.) chemotherapy. Intra-operative i.p. chemotherapy is an interesting method of administration by enhancing the diffusion of chemotherapy. This study had assessed the feasibility of intra-operative i.p. chemotherapy in patients with peritoneal carcinoma of ovarian cancer.

**Methods:**

From January 2003 to February 2006, 47 patients with stage III ovarian cancer were treated with standard paclitaxel carboplatin intravenous chemotherapy and debulking surgery with intra-operative i.p. chemotherapy. After optimal cytoreductive surgery, defined by no unresectable residual disease > 1 cm, i.p. chemotherapy was performed during surgery. The peritoneal cavity was filled by 3 litres of isotonic saline pre-heated at 37 degrees and 90 mg of cisplatin. The sequence was repeated twice during 2 hours based on previous published studies which optimized the cisplatin dosage and exposure duration. Optimal diffusion was obtained by stirring by hands during the 2 hours.

**Results:**

Median age was 59.6 years. No severe haematological or non-haematological toxicity induced by intra operative i.p. chemotherapy was reported. No patient died due to the complications of surgery or the i.p. chemotherapy. No neurotoxicity occurred, and one patients had renal impairment.

**Conclusion:**

This study demonstrates the feasibility of intra-operative i.p. chemotherapy with cisplatin after optimal resection of peritoneal tumor nodules. Further randomized trials are planned to investigate the clinical benefit of this therapeutic modality.

## Background

Ovarian cancer is the leading cause of gynaecologic cancer-related death in most industrialized countries and the fifth cause of cancer death among women [1-3]. Approximately 60% of women have an advanced FIGO stage III-IV ovarian cancer at diagnosis and the 30% 5-year survival rate is dramatically poor. The peritoneal cavity is the main site of disease involvement in ovarian cancer [4,5]. Standards treatments include exploratory laparotomy with cytoreductive surgery followed by intra-venous (i.v.) platinum/taxane-based chemotherapy [6-8]. Nevertheless, additional intra-peritoneal (i.p.) chemotherapy is an encouraging treatment option for ovarian cancer with peritoneum involvement [9,10]. The rationale for i.p. chemotherapy is based on high drug concentration exposure in the peritoneal cavity leading to an increased cytotoxicity and avoiding a high level of systemic toxicity [11-16]. However, despite the advantage of a high concentration of anticancer drugs, the results obtained with i.p. chemotherapy are still debatable in terms of complete and lasting responses [17]. One of the reasons suggested to explain those failures was the difficulty for i.p. chemotherapy to diffuse widely in the peritoneal cavity due to adhesion and/or anatomic niches. Intra-operative i.p. chemotherapy was suggested with the aim to improve its results [18,19]. The administration of i.p. chemotherapy during surgery allows an optimal peritoneal cavity exposure controlled by the surgeon who stirs the cisplatin containing solution by hand. The goal of this present report was to analyze the feasibility and the toxicity of this method of intra-operative i.p. chemotherapy.

## Patients and methods

Between January 2003 and February 2006, 47 patients with advanced epithelial ovarian cancer classified FIGO stage IIIC were included and treated in our institution, University Hospital of Besançon (France). In 31 patients, treatment consisted in 4 cycles of induction i.v. chemotherapy with 175 mg paclitaxel per square meter of body surface area (mg/m^2^) over 3 hours and area under the (AUC) curve targeted to 5 for carboplatin over 30 minutes on day 1, every 3 weeks. This chemotherapy was followed by debulking surgery with intra operative i.p. chemotherapy using cisplatin. Initial debulking surgery was performed when complete tumoral resection seemed feasible. In 31 cases this initial surgery seemed not possible and induction chemotherapy was administered. In 16 patients initial debulking surgery was performed and appeared to be suboptimal with major residual lesions in 7 cases. In all cases, complementary systemic chemotherapy with paclitaxel and carboplatin was administered. This chemotherapy was followed by a second look surgery with or without tumoral debulking and with the administration of intraperitoneal chemotherapy if residual disease was smaller than 10 mm. After debuloking surgery, 2 to 4 additional cycles of paclitaxel/carboplatin chemotherapy were administered. In 16 patients, treatment included initial debulking surgery followed by 6 to 8 cycles of i.v. paclitaxel/carboplatin chemotherapy. Then, second-look surgery with intra-operative i.p. ciplatin chemotherapy was performed. In all cases, an optimal cytoreductive surgery, defined by no unresectable residual disease larger than 10 mm, was achieved either at presentation or after completion of induction chemotherapy. Intraoperative i.p. chemotherapy was performed according to Royer et al description using the optimization suggested by pharmacokinetics analysis [20,21]. These authors have identified an optimal dose and duration of exposure of cisplatin for intraperitoneal treatment based on pharmacokinetic – pharmacodynamic studies. Previously, preclinical results have identified the minimal doses and the optimal duration of platin exposure required to obtain maximal cytotoxic activity [22]. During surgery, after resection of all residual disease, the peritoneal cavity was filled by 3 liters of isotonic saline pre-heated at 37° and a total cisplatin dose of 90 mg during 1 hour. The sequence was repeated twice and the optimal distribution was obtained by stirring by hand during the 2 hours. Then, the peritoneal cavity was cleared out and rinsed before closing down. Concomitant i.v. hydration with 3000 ml normal saline, 2.2 mM Ca^2+ ^glucuronate, 1 g/l Mg^2+^, 2 g/l KCl and 3 g/l NaCl was administered to prevent renal toxicity. The follow up of the patients was performed every 12 weeks with clinical examination, CA 125 test, abdominal and pelvic ultrasound or CT scan.

### Statistical analysis

All estimated confidence interval parameters were designed with a significance level of α = 0.05. Time-to-event endpoints of overall survival (OS), disease progression (DP) and response duration curves were evaluated using Kaplan-Meier non-parametric methods [23] using JMP^® ^Software (SAS, Cary NC). The duration of DP is defined as the time from the diagnosis to the date of progressive disease or death. The overall survival is defined as the time from the diagnosis to date of death or last follow-up.

## Results

### Patients and treatment

Data were updated October 27, 2006. Table 1 summarizes the characteristics of patients. A majority of patients (66%) had initial chemotherapy followed by debulking surgery. The median duration of surgery was 7 hours and 10 minutes (range: 5 hours 10 minutes to 9 hours and 30 minutes) including the 2 hours of the administration of i.p. chemotherapy (table 2). The median hospitalization duration in intensive care unity and surgery unity was 3 days (range: 1 to 21 days) and 18 days (range: 12 to 66 days), respectively. The median delay between the surgery and the resumption of feeding was 7 days (range: 3 to 28 days). Rehospitalization in the surgery unit was required for 16 patients (median: once, range: 0 to 3 times), for restoring bowel continuity (in 7 patients), infection (3 patients), abdominal pain (3 patients), bowel occlusion (2 patients) and renal failure (1 patients).

**Table 1:** Characteristics of patients

**Characteristics of patients (N = 47)**	
**Median age (years)**	59 (Range: 35–75)

**GOG Performans status 0**	40 (85%)
**≥ 1**	7 (15%)

**Histologic type**	
***Serous adenocarcinoma***	
Well differentiated	19 (40%)
Moderately/poorly differentiated	20 (43%)
***Endometrioid adenocarcinoma***	5 (11%)
Mixed epithelial carcinoma	2 (4%)
Clear cell carcinoma	1 (2%)

**Visible residual macroscopic disease**	
Yes	20 (43%)
No	27 (57%)

**Induction Chemotherapy (paclitaxel – carboplatin regimen)**	31 (66%)

**Initial surgery**	16 (44%)

**Second look surgery**	16/16* (100%)

**Total number of i.v. cycles of chemotherapy**	6 (Range:5–8)

* Surgery during which was performed intra operative i.p. chemotherapy

**Table 2:** Duration of hospitalization

	**Median**	**Range**
Duration of surgery* (h = hours, mn = minutes)	7 h 10 mn	5 h 10 mn- 9 h 30 mn

**Duration of hospitalization in intensive care unity**	3 Days	1–21 Days

**Duration of hospitalization in surgical unity**	18 Days	12–66 Days

**Delay between surgery and resumption of feeding**	7 Days	3–28 Days

**Number of rehospitalization**	1**	0–3 times

* including the 2 hours of IIP chemotherapy

** concerning 16 patients

### Complications and toxicity

The safety analysis reported no severe haematological or non-haematological toxicity induced by intra operative i.p. chemotherapy. No patient died due to the complications of surgery or the i.p. chemotherapy. The most frequent complication was infection, including urinary or pulmonary infection which occurred in 9 and 3 patients, respectively (table 3). 13 of 47 patients require a re-laparotomy (4 patients needed 2 re-laparotomy) and we exclude from this total the 6 patients who had a surgical restoration after 30 days. Peritonitis and intra-abdominal abscess was observed in 5 and 3 patients respectively, they required a laparotomy to rinse and clean up the peritoneal cavity. This surgical intervention was also necessary for intra abdominal bleeding and intestinal necrosis which occurred in 7 and 2 patients, respectively. Two patients presented a bowel occlusion which recovered with medical treatment. Thromboembolic events occurred in 5 patients, including a pulmonary embolism in 1 patient. In 5 patients, grade 2 renal failures occurred during the first 10 days after surgery with i.p. cisplatin chemotherapy and they recovered after i.v. hydration with normal saline 2.2 mM Ca^2+ ^glucuronate, 1 g/l Mg^2+^, 2 g/l KCl and 3 g/l NaCl. One patient presented a grade 3 renal failure with uncompleted recovery and which needed stringent follow up. No grade 4 haematological toxicity was observed. Six patients presented grade 3 anaemia and 5 patients presented grade 3 neutropenia without fever. Grade 2 thrombopenia occurred in only one patient. After a 30-day period, complications were surgery related (table 4). Chronic diarrhea, dysuria and abdominal pain were observed in 9, 8 and 2 patients, respectively. A surgical restoration was necessary for vesico-vaginal fistula, bowel fistula and entero-vesical fistula in 3, 2 and 1 patients, respectively.

**Table 3:** Early complication and toxicity by patient including intraoperative toxicity (within 30 days after surgery and i.p. chemotherapy)

**Type of complication**	**Number of patients**	**% of patients**
**Post operative pain**	10	21.3

**Infectious Peritonitis**	5	10.6

**Intraabdominal abscess**	3	6.4

Others infectious complications*	12	25.5

**Intraabdominal bleeding**	7	14.9

**Intestinal necrosis**	2	4.3

**Bowel occlusion**	2	4.3

Thromboembolic events**	5	10.6

**Renal failure**		
**Grade 1**	6	12.8
**Grade 2**	5	10.6
**Grade 3**	1	2.1
**Grade 4**	0	0

**Anaemia grade 3**	5	10.6

**Neutropenia grade 3**	4	8.5

**Febrile Neutropenia**	0	0

**Thrombopenia grade 2**	1	2.1

* including 9 urinary infection and 3 pulmonary infection

** including: deep venous thrombosis of the leg and the arm in 3 and 1 patients respectively and a pulmonary embolism in 1 patient.

**Table 4:** Complication's later than 30 days since surgery and i.p. chemotherapy

**Type of complication**	**Number of patients**	**% of patients**
**Vesico-vaginal fistula**	3	6.4

**Bowel fistula**	2	4.3

**Entero-vesical fistula**	1	2.1

**Chronic diarrhea**	9	19.1

**Chronic dysuria**	8	17

**Chronic abdominal pain**	2	4.3

Loss of weight *	4	8.5

* more than 10% of the weight 3 months after surgery

### Efficacy

**Figure 1:**
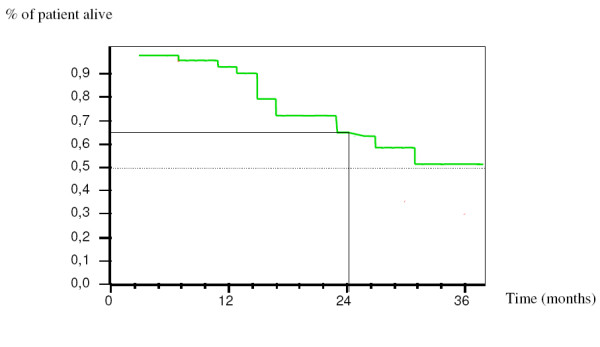
**Disease free survival in intraoperative i.p. chemotherapy group and control group**.

After a median follow up of 23.3 months, a recurrence of the disease was observed in ten patients. The median disease free progression duration was 14.3 months (range: 9.6 – 23.3 months). Sites of relapse were peritoneal carcinomatosis in 4 patients, peritoneal nodes in 4 patients, pleural effusion in 3 patients, liver metastasis in one patient. At 24 months, the rate of patients alive without recurrence was 62.5% [95% CI, 55% to 70%] (Figure 1). No data in term of OS was of value due to the length of follow-up.

## Discussion

Intra-peritoneal administration of chemotherapy is commonly performed at a distance from surgery by an i.p. catheter with artificial ascites [9,24]. Women who have a successful optimal resection of their cancers with microscopic residual tumour and no bowel resection are the best candidates for i.p. chemotherapy [25]. It seems favorable if possible to perform a supra-cervical hysterectomy and not to enter the vagina because when the vagina is opened leakage of chemotherapy via the vagina is mostly risked. However, even if there is no absolute contraindication to placement of this access device, complications could occur such as catheter infection or intra-abdominal abscess, bowel injuries, kinking of the catheter or inflow obstruction and leakage of chemotherapy around the port or into the surrounding subcutaneous tissue [24]. Abdominal pain is the most common i.p. chemotherapy-related risk. In most cases it is due to the distension of the abdomen but it is very important not to underestimate the risk of peritonitis or bowel injuries which is a medical and surgical emergency. Others i.p. chemotherapy complications included those linked to the drug administered. The most frequently used drug is cisplatin [26-30]. Nausea, vomiting and renal toxicity must be prevented by effective anti-emetics drugs and suitable i.v. hyperhydration considering the systemic exposure to the agent after i.p. comparable to i.v. administration [20]. Since the emergence of this concept of i.p. administration with chemotherapy reported by Dedrick et al in 1978 [31], several phase II studies have confirmed the favorable trends obtained by these treatments in terms of overall and/or progression – free survival [32,33]. Comparison between i.p. and i.v. treatments was undertaken in several randomized phase III clinical trials [26-28]. Recently, Armstrong et al [28] reported a highly significantly improvement in progression-free (24 months versus 18.3 months; p = 0.027) and overall survival (65.6 months versus 49.7 months; p = 0.017) with i.p. therapy. Because of the need to recover from surgery, the beginning of the i.p. chemotherapy is often delayed and performed at distance of surgery. The occurrence of adhesion barriers will be embarrassing for an optimal distribution of chemotherapy in the abdominal cavity. One should consider that the most frequent sites of recurrences are those where i.p. chemotherapy is unable to reach. Advocates of adhesion formation barriers could be used to limit their incidence but efficacy is not clearly demonstrated in this situation. Relying on these considerations, i.p. chemotherapy during surgery was suggested with the aim to improve results. The administration of i.p. chemotherapy during the surgery allows an optimal peritoneal cavity exposure warranted by the control of the surgeon who stirs the cisplatin liquid by hand inside the peritoneal cavity. This method presents the advantage to not require an i.p. catheter and to provide optimal diffusion. The aim of our study was to analyze its feasibility. One of our questions was to determine the optimal dose of i.p. cisplatin, knowing that standard regimen in i.p. clinical trial uses a dose of 50 to 100 mg/m^2 ^cisplatin [24]. A search for optimizing the dose and schedule of intraperitoneal cisplatin was performed, with the aim to increase the intraperitoneal concentration and to limit the systemic spread. The addition of epinephrin in preclinical model have shown to achieve this goal and warrant further clinical studies [22]. The dose used in the present study issued from studies performed in 2005 regarding serum and i.p. pharmacokinetics of platin with intra-operative chemotherapy. This study concluded that cisplatin administered at the dose of 50 mg/m^2 ^during 2 hours i.p. chemotherapy resulted in an early dramatic decrease in i.p. drug concentration, below the targeted threshold for activity within 15 minutes. The author suggested that performing twice 1 hour i.p. cisplatin chemotherapy should increase the length of peritoneal exposure to a local cytotoxic dose [21]. Relying on these results, we proposed in our study to perform i.p. chemotherapy in 2 consecutive one-hour administrations with cisplatin given at a dose of 90 mg. The present paper is the first demonstration of the feasibility for this modality of intra-operative i.p. chemotherapy. The described modalities of administration for i.p. chemotherapy enhance the distribution of cisplatin in the peritoneal cavity without inducing severe and non-manageable toxicities. Those results suggest further randomized clinical studies aimed to establish its benefit in terms of survival.

## Competing interests

The authors declare that they have no competing interests.

## Authors' contributions

EG conceived and participated to the design of the study, included patients, performed data analysis and interpretation, followed-up patients and write the manuscript DD included patients, performed surgery and intra-peritoneal chemotherapy, followed-up patients and participate to the data collection BH included patients, performed surgery and intra-peritoneal chemotherapy and followed-up patients MC performed intra-peritoneal chemotherapy and followed-up patients VL included patients, followed-up patients and participate to the data collection MD included patients, followed-up patients and participate to the data collection US included patients, followed-up patients and participate to the data collection BR conceived and participated to the design of the study, participated to interpretation and the writing of the manuscript BC conceived and participated to the design of the study, participated to data analysis, interpretation and the writing of the manuscript XP conceived and participated to the design of the study, participated to the writing of the manuscript, performed data analysis, statistical analysis and the interpretation All authors read and approved the final manuscript.
